# Plasma Levels of Acyl-Carnitines and Carboxylic Acids Correlate With Cardiovascular and Kidney Function in Subjects With Sickle Cell Trait

**DOI:** 10.3389/fphys.2022.916197

**Published:** 2022-07-13

**Authors:** Travis Nemkov, Sarah Skinner, Mor Diaw, Saliou Diop, Abdoulaye Samb, Philippe Connes, Angelo D’Alessandro

**Affiliations:** ^1^ Department of Biochemistry and Molecular Genetics, University of Colorado Denver, Aurora, CO, United States; ^2^ Inter-university Laboratory of Biology of Motor Function EA7424, Vascular Biology and the Red Blood Cell Team, Claude Bernard University Lyon 1, Lyon, France; ^3^ Laboratory of Physiology and Functional Exploration, FMPO, UCAD, Dakar, Senegal; ^4^ IRL3189 Environnement, Santé, Sociétés CNRS/UCAD Dakar/ UGB Saint-Louis/ USTT Bamako/ CNRST Ouagadougou, Dakar, Senegal; ^5^ Laboratory of Hemato-immunology, FMPO, UCAD, Dakar, Senegal

**Keywords:** metabolome, sickle cell trait, hemoglobin S, lands cycle, mitochondria

## Abstract

Subjects with sickle cell trait (SCT) carry one copy of mutated β-globin gene at position E6V at the origin of the production of sickle hemoglobin (HbS). Indeed, individuals with SCT have both normal hemoglobin and HbS, in contrast to patients with sickle cell disease who inherited of two copies of the mutated gene. Although SCT is generally benign/asymptomatic, carriers may develop certain adverse outcomes such as renal complications, venous thromboembolism, exercise-induced rhabdomyolysis … However, little is known about whether similar metabolic pathways are affected in individuals with SCT and whether these metabolic derangements, if present, correlate to clinically relevant parameters. In this study, we performed metabolomics analysis of plasma from individuals with sickle cell trait (*n* = 34) compared to healthy controls (*n* = 30). Results indicated a significant increase in basal circulating levels of hemolysis markers, mono- (pyruvate, lactate), di- and tri-carboxylates (including all Krebs cycle intermediates), suggestive of systems-wide mitochondrial dysfunction in individuals with SCT. Elevated levels of kynurenines and indoles were observed in SCT samples, along with increases in the levels of oxidative stress markers (advanced glycation and protein-oxidation end-products, malondialdehyde, oxylipins, eicosanoids). Increases in circulating levels of acyl-carnitines and fatty acids were observed, consistent with increased membrane lipid damage in individuals with sickle cell trait. Finally, correlation analyses to clinical co-variates showed that alterations in the aforementioned pathways strongly correlated with clinical measurements of blood viscosity, renal (glomerular filtration rate, microalbuminuria, uremia) and cardiovascular function (carotid-femoral pulse wave velocity, blood pressure).

## 1 Introduction

Sickle cell disease (SCD) was the first identified molecular disease (Adebiyi et al., 2019). A single amino acid substitution (hemoglobin beta—HBB E6V in the mature peptide after N-terminal excision) is the etiological genetic factor underlying SCD (Ingram, 1959). This mutation impacts hemoglobin allostery and polymerization in response to hypoxia, a phenomenon underlying the so-called sickling of the red blood cells (RBCs) (Ghatge et al., 2016). SCD patients carry two copies of this beta globin variant, and the primary hemoglobin present in their RBCs is called sickle hemoglobin (HbS). Incidence of SCD is high in people whose ancestors come from sub-Saharan Africa, Spanish speaking regions (South America, Cuba, Central America), Saudi Arabia, Oman, India, and Mediterranean countries such as Turkey, Greece, and Italy (John, 2010). Individuals who are heterozygous for the normal beta globin gene (HbA) and one copy of HbS are referred to as having sickle cell trait (SCT). These individuals do not show classic symptoms of SCD, and are generally asymptomatic. However, SCT is associated with a range of clinical complications, including increased urinary tract infection in women, gross hematuria, complications of hyphema, splenic infarction with altitude hypoxia or exercise, life-threatening complications of exercise, exertional heat illness (exertional rhabdomyolysis, heat stroke, or renal failure) or idiopathic sudden death (John, 2010). According to the Center for Disease Control, the incidence estimate for SCT in the United States was 73.1 cases per 1,000 black newborns, 3.0 cases per 1,000 white newborns, and 2.2 cases per 1,000 Asian or Pacific Islander newborns. The incidence estimate for Hispanic ethnicity (within 13 states) was 6.9 cases per 1,000 Hispanic newborns (Ojodu et al., 2014).

RBC metabolic reprogramming in response to hypoxia facilitates oxygen off-loading to sustain increased oxygen demand during exercise (Nemkov et al., 2021) or to promote acclimatization upon exposure to high-altitude hypoxia (D'Alessandro et al., 2016). Small molecules like high-energy phosphate compounds, 2,3-diphosphoglycerate (DPG) and adenosine triphosphate (ATP) allosterically regulate hemoglobin by stabilizing the tense deoxygenated state (Yuan et al., 2015) and promoting oxygen off-loading (Donovan et al., 2021). Other signaling cascades at the metabolic level have been described in recent years as critical regulators of RBC physiological and pathological responses to hypoxia (D'Alessandro and Xia, 2020), including (i) increases in the levels of plasma adenosine (Liu et al., 2016), and (ii) sphingosine 1-phosphate (Sun et al., 2016); (iii) stabilization of the enzyme bisphosphoglycerate mutase by post-translational modification via RBC transglutaminase 2 (Xu et al., 2022); (iv) rewiring of RBC glucose oxidation from the pentose phosphate pathway (at high O_2_ saturation) to glycolysis (at low O_2_ saturation) as a strategy to boost the generation of ATP and DPG at the expense of the capacity to generate reducing equivalents to sustain antioxidant reactions (i.e., NADPH) (Reisz et al., 2016; Issaian et al., 2021); (v) further stabilization of deoxyhemoglobin at the membrane through binding to the N-terminus cytosolic domain of band 3, the most abundant membrane protein in RBCs (Lewis et al., 2009). All of these metabolic pathways are critical to RBC physiological responses to hypoxia, but are detrimental in the context of SCD, where stabilization of the deoxygenated form of HbS favors polymerization and sickling (Zhang et al., 2011; Sun et al., 2015; Wu et al., 2016; Sun et al., 2017a). For example, studies have shown that adenosine signaling through receptor A2b favors a metabolic switch to glycolysis, DPG generation and sickling, a cascade that involves the activation of adenosine monophosphate-dependent kinase (AMPK) and protein kinase A (PKA) (Zhang et al., 2011). PKA-dependent activation of RBC sphingosine kinase 1 stimulates the synthesis of S1P (Sun et al., 2015), which in turn cooperates with DPG in the stabilization of the Tense deoxygenated state of HbS (Sun et al., 2017a). Of note, incomplete erythropoiesis has been reported to favor retention of functional mitochondria in mature SCD erythrocytes (Tumburu et al., 2021). Circulating mitochondrial DNA is a proinflammatory damage-associated molecular pattern (DAMP) in SCD (Tumburu et al., 2021). Compromised oxygen kinetics in SCD are in part explained by altered vasodilatory function as a result of impaired arginine metabolism and nitric oxide synthesis (Gladwin et al., 2011; Morris et al., 2020). Mitochondrial dysfunction in peripheral tissues and mature sickle RBC-containing mitochondria are associated with elevated oxidative stress and compromised antioxidant function in SCD (Chirico and Pialoux, 2012; Voskou et al., 2015). To tentatively cope with this phenomenon, trials have been designed involving the supplementation of glutamine (Niihara et al., 2018) as a substrate to fuel the synthesis of glutathione, the main RBC antioxidant. Elevated oxidative stress in SCD targets the membrane lipid fraction, activating a pathway referred to as the Lands cycle for the tentative repair of damaged lipids through activation of phospholipase A2 enzymes to cleave oxidatively-damaged fatty acyl components, and transfer of acyl-moieties from CoA-conjugates (in equilibrium with acyl-carnitine pools) to lysophospholipids (Wu et al., 2016). Of note, similar metabolic adaptations have been reported in RBCs in response to oxidative stress in chronic kidney disease (Xu et al., 2022), acute kidney dysfunction (Bissinger et al., 2021), COVID-19 (Thomas et al., 2020a) or in response to exercise-induced vesiculation (Nemkov et al., 2021). Correction of these pathways—including fatty acid oxidation and acyl-carnitine metabolism (Culp-Hill et al., 2018) - have been reported in SCD recipients upon exchange transfusion therapy. Altered antioxidant metabolism and increased lipid peroxidation contribute to a higher susceptibility to hemolysis of SCD RBCs. Increased susceptibility to hemolysis in SCD is an etiological contributor to kidney and pulmonary dysfunction and inflammatory complications in patients with SCD (Drawz et al., 2016; Page et al., 2021) and animal models (Buehler et al., 2021). However, subjects with SCT have no hemolysis in the absence of perturbations and have milder/no clinical manifestations.

Several large scale population-based studies including African American participants have demonstrated a 1.5 to 2.0 fold greater risk of chronic kidney disease in individuals with SCT compared to non-carriers (Naik et al., 2014; Naik et al., 2017). The hypoxic and acidotic environment of the kidney could promote repeated sickling of SCT RBCs, even under unstressed physiologic conditions, that may cause permanent tissue damages. In addition, SCT has been established as a modest risk factor of venous thromboembolism among African Americans, but this risk is 1.5 to 2.0 fold greater in those with SCT compared to those without (Austin et al., 2007; Folsom et al., 2015). While the mechanisms of metabolic reprogramming have been extensively investigated in humans (Darghouth et al., 2011; Zhang et al., 2011; Wu et al., 2016) and mouse models of SCD (Dembélé et al., 2020), to the best of the authors’ knowledge, literature is scarce or non-existent in the context of SCT, which is the focus of the present study.

## 2 Methods

### 2.1 Cohort enrollment

Subject recruitment and sample collection took place at the Cheikh Anta Diop University in Dakar, Senegal (Western African country) from August to October 2017. In total, 64 subjects were recruited from the National Center of Blood Transfusion and from the general population of Dakar. All participants were Senegalese adults (48.56 ± 6.42 years old; BMI of 23.44 ± 3.37; 10 male/54 female). Subjects were screened for SCT and assigned to the control or SCT groups.

SCT status was screened by isoelectric focusing, citrate agar electrophoresis, hemoglobin fraction quantification (high-performance liquid chromatography), and a solubility test. Individuals who smoked as well as women who were pregnant or using oral contraception were excluded from the study. The study protocol was conducted in accordance with the Declaration of Helsinki and was approved by the Ethics Committee of Cheikh Anta Diop University (reference: 0221/2016/ CER/UCAD). All subjects gave informed, written consent.

### 2.2 Blood sampling and Clinical Measurements

Subjects arrived at the Laboratory of Medical Physiology (Cheikh Anta Diop University) at 8:00 A.M for clinical assessments, including confirmed negative presence of malaria by rapid immunochromatographic test. All of the subjects were in a fasted state and were instructed to refrain from physical activity for 24 h before the visit. Blood was drawn into heparin tubes for lipid measurement, fluoride tubes for glucose measurement and EDTA tubes for analyses of hemoglobin A1c (HbA1c), blood rheology, metabolomics, cytokines, and oxidative stress and antioxidant enzyme capacity. The blood samples used to evaluate metabolomic and lipidomic profiles, and to measure oxidative stress and antioxidant enzyme capacity were immediately centrifuged, and the plasma was then stored at -80°C until analyses were conducted.

Blood pressure and pulse wave velocity measurements were conducted as previously described (Skinner et al., 2018). Briefly, systolic blood pressure (SBP) and diastolic blood pressure (DBP) were measured in the left arm using a manual sphygmomanometer while the subject was in a seated position (Omron M3; Intellisense, Kyoto, Japan). The aortic distensibility, carotid-radial and carotid-femoral pulse wave velocity (PWV) were measured using two pressure-sensitive transducers with an automated system (Pulse Pen; DiaTecne, Milan, Italy).

Urinary albumin concentration (UAC) was measured using early morning spot urine (HemoCue Albumin 201 System). Serum creatinine level was measured using the standard Jaffe method. The estimated glomerular filtration rate (eGFR) was calculated using the Chronic Kidney Disease Epidemiological Equation, with the appropriate corrections for race and sex. Subjects with an eGFR <60 ml/min/1.73 m^2^ were classified as having reduced renal function (moderately or severely decreased eGFR).^40^e

### 2.3 Biochemical Parameters

Fasting glucose was measured using an enzymatic glucosidase-peroxidase method (Urit Medical Electronic Co., Guilin, People’s Republic of China). HbA1c was measured using capillary electrophoresis on a Capillary 3 Tera device (Sebia, Lisses, France). Plasma lipids (triglycerides, total cholesterol, HDL cholesterol, and LDL cholesterol) were evaluated using standard enzymatic methods.

### 2.4 Hemorheological and Hematological Parameters

Hematocrit was measured after blood microcentrifugation. Other hematological measures were done using a hematological analyzer. Plasma viscosity was measured at 37°C using a cone-plate viscometer at a shear rate of 375 s^−1^. Oxygenated whole blood at native hematocrit was used to measure whole blood viscosity at varying shear rates (5.62, 11.25, 22.5, 45, 90, 225, and 375 s^−1^), at 37°C, using a cone-plate viscometer (Pro DV-II+, with CPE40 spindle; Brookfield, Middleboro, MA).

### 2.5 Oxidative stress markers, AGEs and Cytokines

Plasma antioxidant activity (plasma superoxide dismutase (SOD), catalase, and glutathione peroxidase (GPX)), and markers of oxidative stress damage (advanced oxidation protein products (AOPP), and malondialdehyde) were determined as previously described (Delrieu et al., 2021) (Delrieu et al., Oxidative Medicine and Cellular Longevity 2021).

ELISA kits were used to measure plasma concentrations of AGEs (Cell Biolabs, Inc. San Diego, CA) and interleukin (IL)-1β (Genetex, Irvine, CA). The Bio-Plex Pro Human Cytokine 8-Plex Immunoassay Kit (Bio-Rad) was used to measure concentrations of IL-6, IL-8, IL-10 and tumor necrosis factor-a (TNF-a) in subjects’ plasma. The assays were performed following the instructions with the kit, and cytokine concentrations were measured using the MAGPIX xPONENT 4.2 System (Luminex Corporation, Austin, TX).

### 2.6 Sample processing and Metabolite Extraction

Plasma (20 microliters) was extracted in 980 μl of methanol:acetonitrile:water (5:3:2, *v/v/v*). After vortexing at 4°C for 30 min, extracts were separated from the protein pellet by centrifugation for 10 min at 18,000 g at 4°C and stored at −80°C until analysis.

### 2.7 Ultra-high-pressure Liquid Chromatography-Mass Spectrometry metabolomics and Lipidomics

Analyses were performed using a Vanquish UHPLC coupled online to a Q Exactive mass spectrometer (Thermo Fisher, Bremen, Germany). Samples were analyzed using a 5 min gradient as described (Nemkov et al., 2017; Nemkov et al., 2019; Reisz et al., 2019). Solvents were supplemented with 0.1% formic acid for positive mode runs and 1 mM ammonium acetate for negative mode runs. MS acquisition, data analysis and elaboration was performed as described (Nemkov et al., 2017; Nemkov et al., 2019; Reisz et al., 2019).

### 2.8 Statistical Analyses

Graphs and statistical analyses (Mann-Whitney non-parametric *U*-test or ANOVA) were prepared with GraphPad Prism 5.0 (GraphPad Software, Inc, La Jolla, CA) and MetaboAnalyst 5.0 (Pang et al., 2021). Spearman correlation analyses and related *p*-values were calculated with MetaboAnalyst 5.0. Experimental design and pathway illustrations were generated using Biorender (www.biorender.com).

## 3 Results

### 3.1 Clinical and Hematological Characterization of the Cohorts of Sickle Cell Trait Carriers and Non-carriers

**FIGURE 1:**
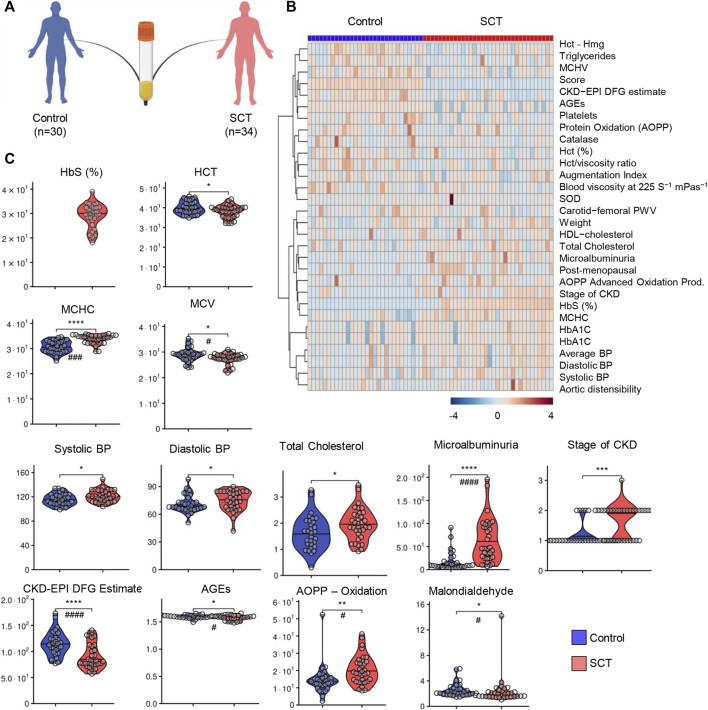
Study design, hematological and clinical parameters. A total of 64 subjects (*n* = 30 in the control group—blue and *n* = 34 in the sickle cell trait—SCT group—red) were enrolled in this study **(A)**. Hierarchical clustering of clinical and hemorheological parameters is shown in panel **(B)**. Violin plots **(C)** show significantly different hematological parameters between the two groups, including % Hemoglobin S (HbS), hematocrit (%), mean cell hemoglobin concentration (MCHC), mean cell volume (MCV); markers of kidey filtration (microalbuminuria) and chronic kidney disease or EPI DFG estimates. Markers of oxidant stress (advances oxidation protein products), total cholesterol levels were also significantly different between the two groups. However, no significant differences were noted with respect to the levels of multiple interleukins (IL-6, IL-8, IL-10 and TNFα—Supplementary Figure S1). Asterisks indicate significance (* *p* < 0.05; ** *p* < 0.01; *** *p* < 0.001; **** *p* < 0.0001 Wilcoxon/Mann-Whitney *U*-test). FDR-corrected *p*-values are provided for all analytes in Supplementary Table S1 and indicated with # using the same significance ranges as per the asterisks in *p*-values above.

A total of 64 subjects (*n* = 30 in the control group and *n* = 34 in the sickle cell trait—SCT group) were enrolled in this study (Figure 1A). Blood from these subjects was characterized for standard hematological parameters, metabolic panels, antioxidant enzyme activity (catalase, superoxide dismutase, glutathione peroxidase), blood viscosity, as detailed in tabulated form in Supplementary Table S1. In addition, all the subjects were characterized for the cardiovascular function, including blood pressure (systolic, diastolic and average), aortic distensibility, carotid-femoral or carotid-radial pulse wave velocity (PWV); markers of kidney function, including stage of chronic kidney disease (CKD), microalbuminuria, Creatinine Equation for Glomerular Filtration Rate (CKD-EPI DFG estimate); weight, age, height, body mass index (BMI), for a total of 60 variables (Supplementary Table S1). Hierarchical clustering of clinical and hemorheological parameters is shown in Figure 1B, which highlights the top 30 variables by significance (unpaired *t*-test). Specifically, we observed significantly different hematological parameters between the two groups, including % Hemoglobin S (HbS), hematocrit (%), mean cell hemoglobin concentration (MCHC), mean cell volume (MCV); markers of kidney filtration (microalbuminuria) and chronic kidney disease or EPI DFG estimates (Figure 1C). Markers of oxidative stress (advances oxidation protein products), total cholesterol levels were also significantly different between the two groups (Figure 1C). However, no significant differences were noted with respect to the levels of multiple interleukins (IL-1, IL-6, IL-8, IL-10 and TNFα—Supplementary Figure S1). In general, individuals with SCT were characterized by lower HCT and MCV, but elevated MCHC. Individuals with SCT also displayed significant increases in the levels of multiple markers of oxidative stress (advanced glycation end-products—AGEs; malondialdehyde; advanced oxidation protein products—AOPP), elevated blood pressure (both diastolic and systolic), higher levels of total cholesterol, increased kidney dysfunction (CKD-EPI DFG, microalbuminuria).

### 3.2 Plasma From Subjects With SCT has Clearly Distinct Metabolic Phenotypes From Healthy Controls, With Specific Pathways Correlating to Clinical Covariates

**FIGURE 2:**
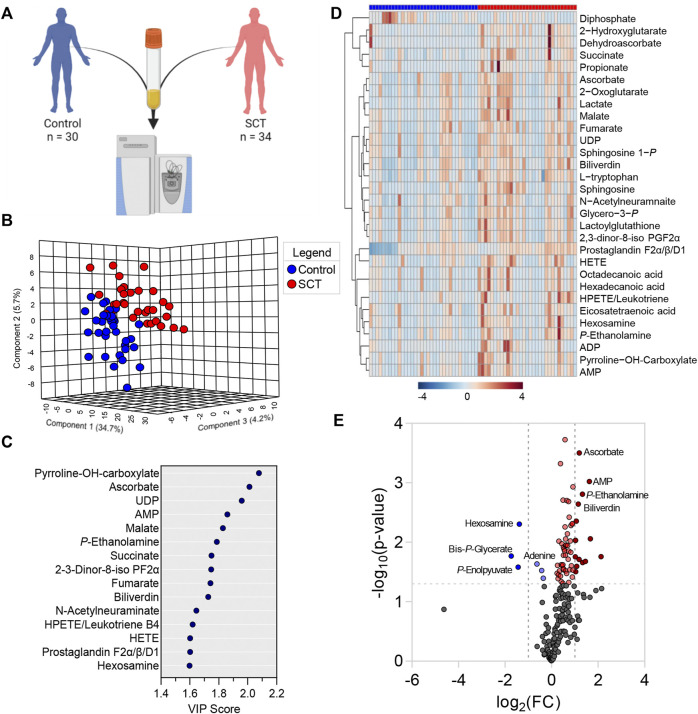
Metabolomics analyses were performed on plasma from healthy controls and subjects with sickle cell trait **(A).** Statistical analyses showed distinct metabolic profiles between the two groups, as gleaned by multivariate principal component analysis **(B)**, variable importance in projection **(C)**, hierarchical clustering analysis **(D)**, and volcano plots **(E)**.

**FIGURE 3:**
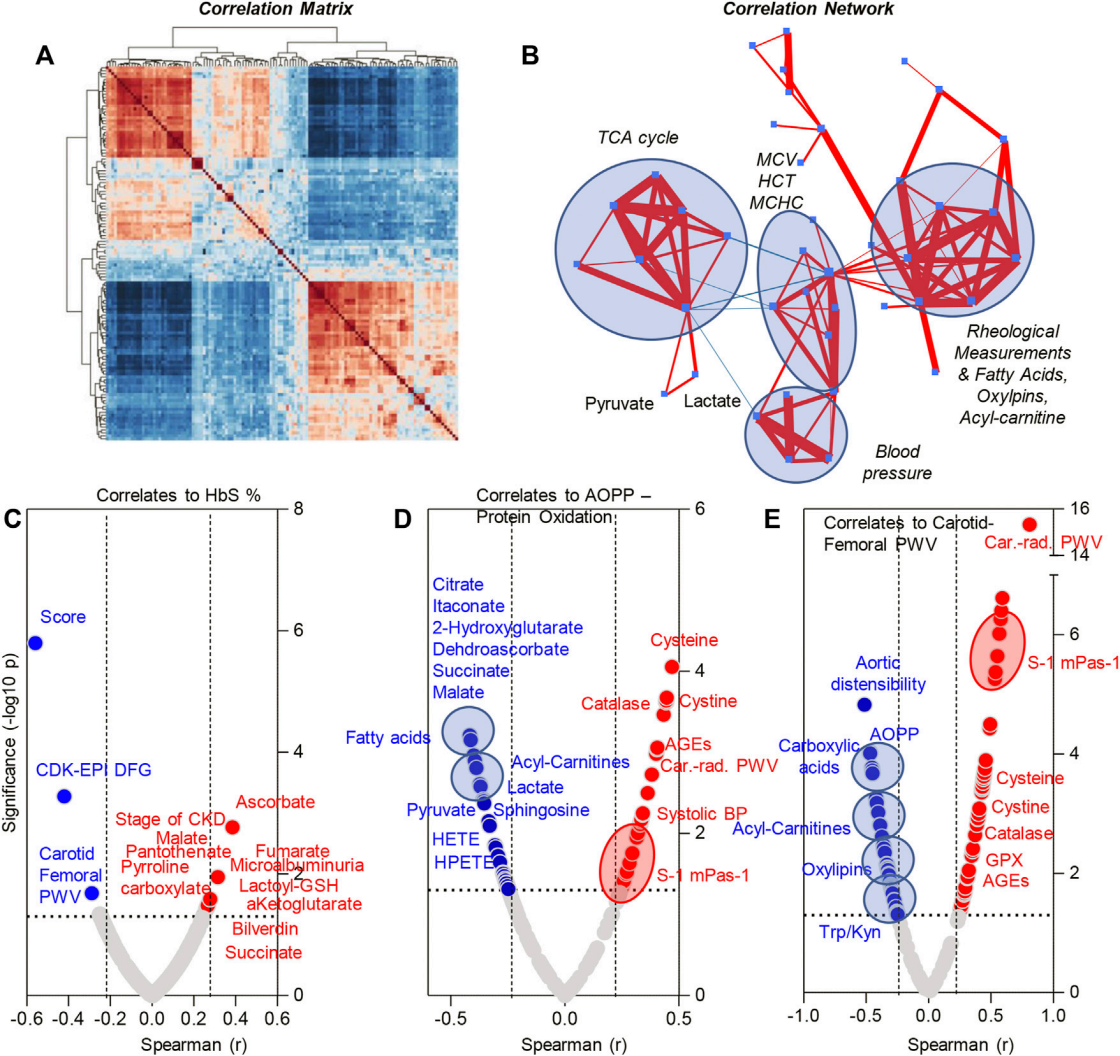
Correlation matrix **(A)**, correlation network **(B)** and metabolic correlates to HbS levels **(C)**, advanced oxidation protein products **(D)** and carotid femoral PWV **(E)**—Spearman correlation.

**FIGURE 4:**
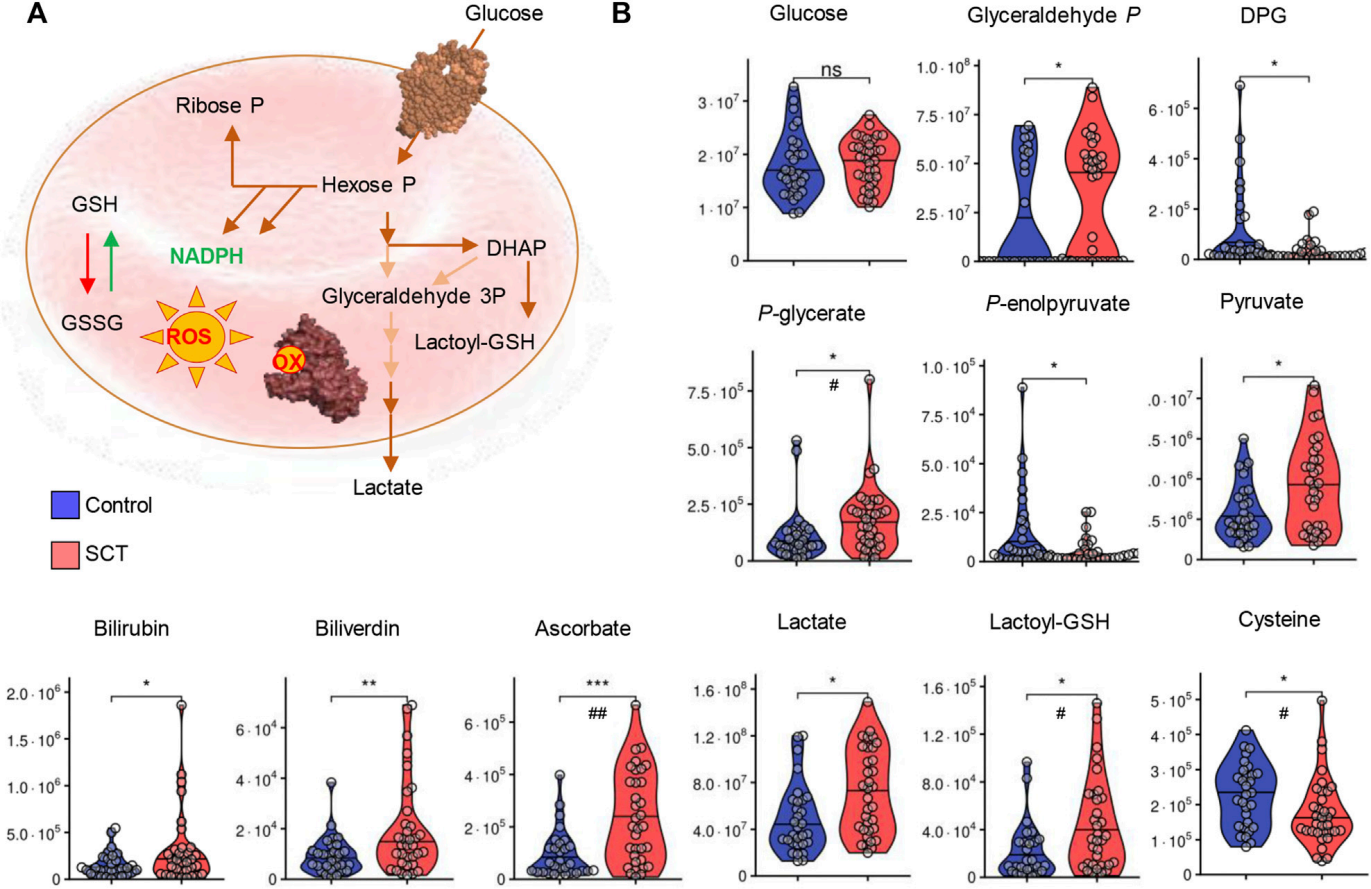
Overview of glycolysis, heme metabolism and redox metabolism in plasma from control (blue) and sickle cell trait (red) subjects.</b> Asterisks indicate significance (* *p* < 0.05; ** *p* < 0.01; *** *p* < 0.001 Wilcoxon/Mann-Whitney *U*-test). FDR-corrected *p*-values are provided for all analytes in Supplementary Table S1 and indicated with # using the same significance ranges as per the asterisks in *p*-values above.

**FIGURE 5:**
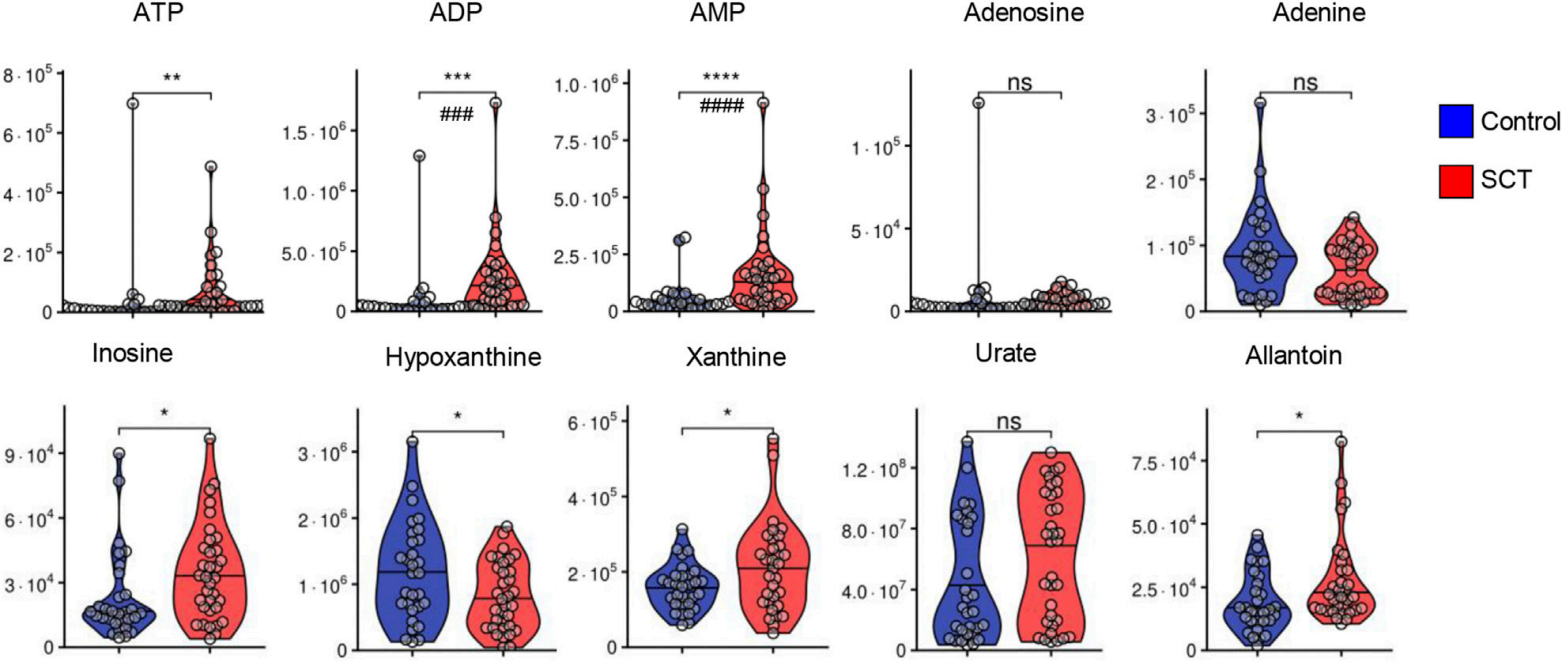
Overview of purine metabolism in plasma from control (blue) and sickle cell trait (red) subjects. Asterisks indicate significance (* *p* < 0.05; ** *p* < 0.01; *** *p* < 0.001; **** *p* < 0.0001 Wilcoxon/Mann-Whitney *U*-test). FDR-corrected *p*-values are provided for all analytes in Supplementary Table S1 and indicated with # using the same significance ranges as per the asterisks in *p*-values above.

**FIGURE 6:**
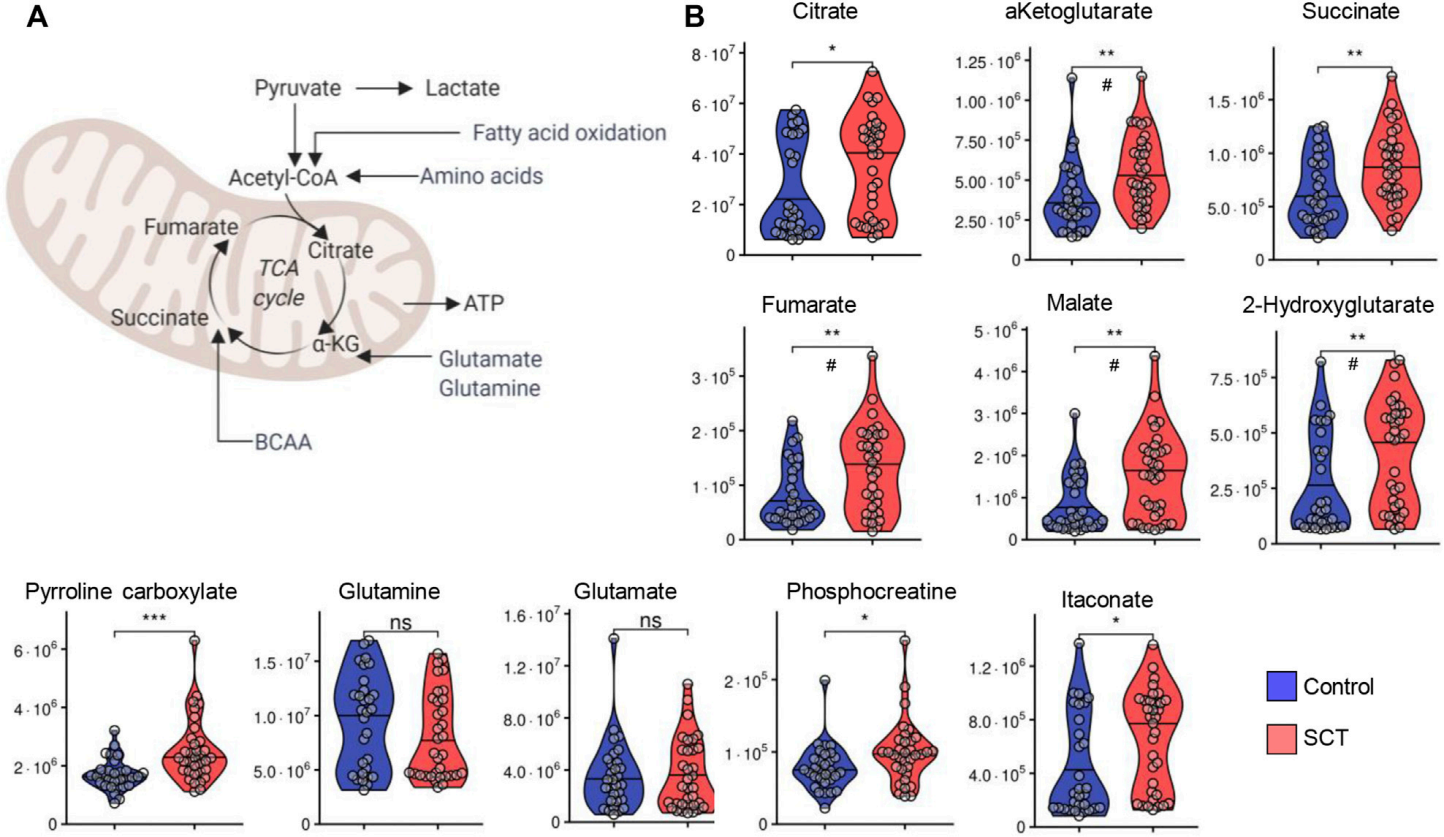
Overview of carboxylic acid metabolism and glutaminolysis **(A)** and violin plot representation of metabolites in these pathways **(B)** as measured in plasma from control (blue) and sickle cell trait (red) subjects. Asterisks indicate significance (* *p* < 0.05; ** *p* < 0.01; *** *p* < 0.001 Wilcoxon/Mann-Whitney *U*-test). FDR-corrected *p*-values are provided for all analytes in Supplementary Table S1 and indicated with # using the same significance ranges as per the asterisks in *p*-values above.

**FIGURE 7:**
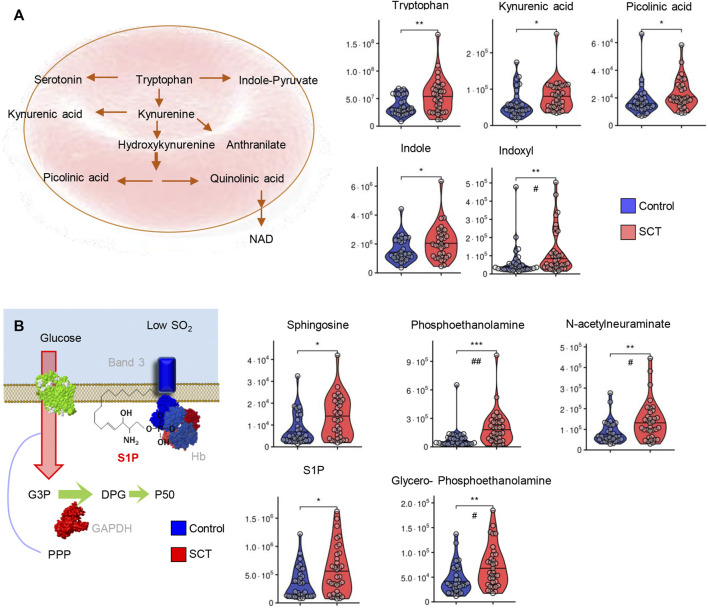
Overview of tryptophan metabolism **(A)** and sphingolipid metabolism **(B)** in plasma from control (blue) and sickle cell trait (red) subjects. Asterisks indicate significance (* *p* < 0.05; ** *p* < 0.01; *** *p* < 0.001 Wilcoxon/Mann-Whitney *U*-test). FDR-corrected *p*-values are provided for all analytes in Supplementary Table S1 and indicated with # using the same significance ranges as per the asterisks in *p*-values above.

**FIGURE 8:**
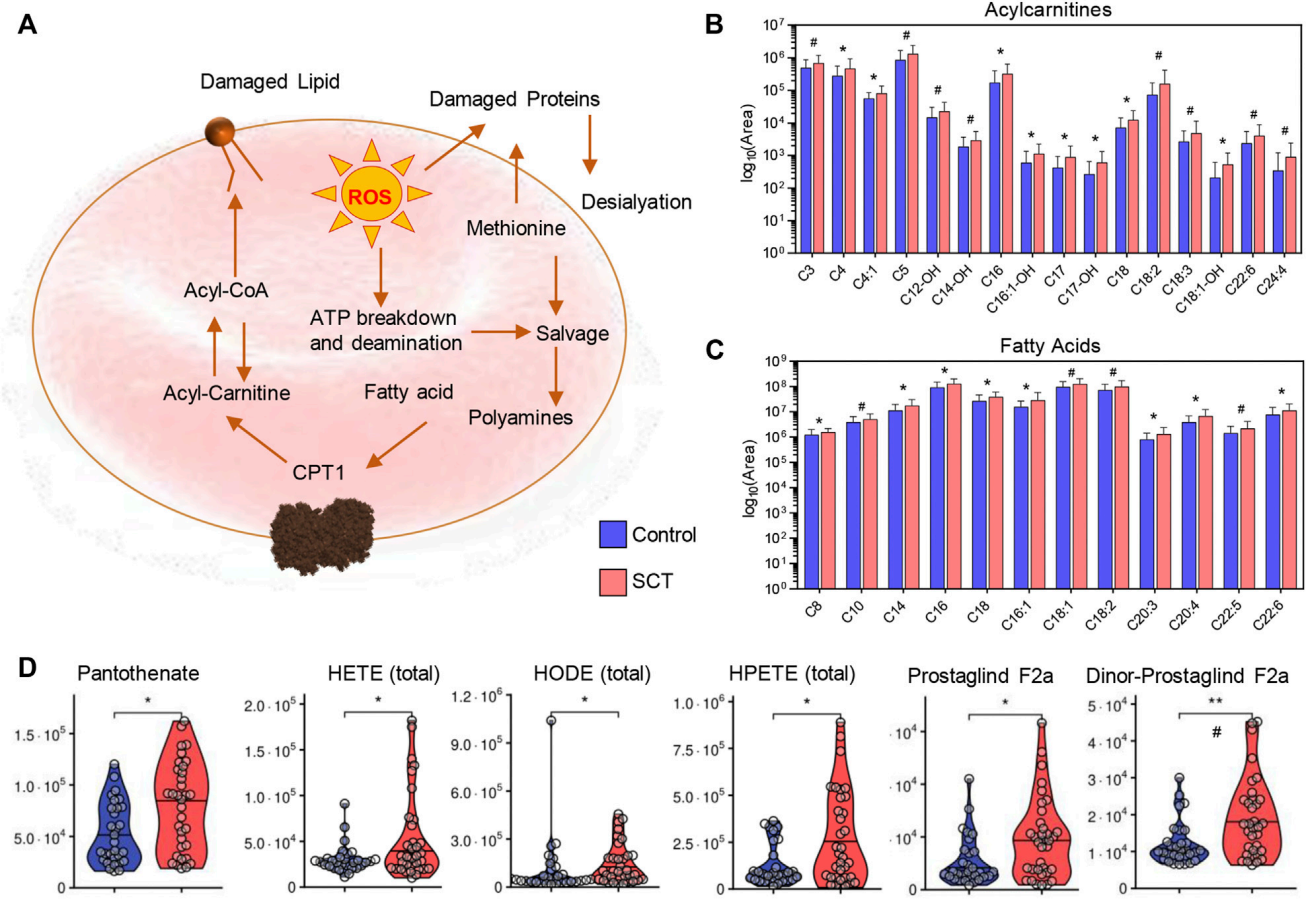
Overview of the Lands cycle **(A)** with a focus on acyl carnitines—**(B)** and free fatty acids—**(C)**, CoA and oxylipin metabolism **(D)** in plasma from control (blue) and sickle cell trait (red) subjects. Asterisks indicate significance (* *p* < 0.05; ** *p* < 0.01; *** *p* < 0.001 Wilcoxon/Mann-Whitney *U*-test). FDR-corrected *p*-values are provided for all analytes in Supplementary Table S1 and indicated with # using the same significance ranges as per the asterisks in *p*-values above.

Metabolomics analyses were performed on plasma from SCT (*n* = 34) and control (*n* = 30) subjects (Figure 2A). Statistical analyses showed distinct metabolic profiles between the two groups, as gleaned by multivariate principal component analysis (PC1 explaining 34.7% of the total variance between the two groups—Figure 2B), variable importance in projection (Figure 2C shows the top 15 metabolites informing clustering of the samples from two groups across PC1), hierarchical clustering analysis (top 30 metabolites by *t*-test are shown in Figure 2D), and volcano plots (Figure 2E). To delve into the potential clinical relevance of these metabolic differences between the two groups, we performed correlation analyses (Spearman) between metabolite levels and non-metabolic measurements (an overview of the correlation matrix resulting from this analysis is provided in Figure 3 A). Correlation values were thus used to generate a network view of the interactions (based on significant correlations) between metabolites and other variables (Figure 3B). In this analysis, each variable is a node, with the highest degree nodes (highest numbers of edges/ significant correlations to other variables) clustering in four main groups: (i) a set of nodes included carboxylic acid metabolites (TCA cycle in Figure 3B), pyruvate and lactate, and measurements of oxidative stress (AOPP, AGEs); (ii) a group of nodes including blood pressure, carotid-femoral or -radial PWV measurements; (iii) a group of nodes including hematological parameters (MCV, HCT, MCHC) and (iv) a group of nodes including rheological measurements (viscosity) and fatty acids, oxylipins and acyl-carnitines (Figure 3B). Based on this analysis, we generated volcano plots highlighting top significant positive (red) or negative (blue) correlates to HbS percentage, AOPP, carotid-femoral PWV (Figures 3.C–E) and CKD-EPI DFG (Supplementary Figure S2). Overall, these correlation analyses identified a strong correlation between glycolytic products (pyruvate, lactate) and carboxylic acids (citrate, alpha-ketoglutarate, 2-hydroxyglutarate, succinate, fumarate, malate, itaconate) and HbS % (positive) and AOPP, carotid-femoral PWV, CKD-EPI DFG (negative); opposite patterns were observed for antioxidant sulfur-containing metabolites (cysteine, cystine, lactoyl-glutathione—lactoyl-GSH), acyl-carnitines, free fatty acids, oxylipins, sphingosine 1-phosphate (S1P). Detailed analyses of how metabolites in these pathways differ between healthy controls and SCT subjects are shown in Figures 4–8.

### ***3.3*** *Mono* ***-, di-*** *and* *Tri* ***-carboxylates*** *Are* *all* *Elevated* *in* *Plasma* *of* ***SCT*** *Subjects*

While metabolomics analyses were performed on plasma, several intracellular metabolites were detectable at significantly higher levels in SCT samples. Specifically, elevated levels of bilirubin and biliverdin in SCT samples are suggestive of increased hemolysis in this group (Figure 4). Elevated circulating levels of ascorbate (vitamin C) and lactoyl-glutathione were accompanied by decreases in the levels of cysteine in SCT plasma compared to controls (Figure 4B). The two groups had comparable glycemic status (Figure 4). Glycolytic metabolites were observed in plasma, with elevations in the levels of triose phosphates (glyceraldehyde 3-phosphate, phosphoglycerate) and decreases in the levels of diphosphoglycerate (DPG), and phosphoenolpyruvate in SCT plasma (Figure 4). Notably, both plasma pyruvate and lactate were significantly higher in SCT plasma (Figure 4A). Circulating levels of high-energy phosphate purines (adenosine tri-, di- and mono-phosphate—ATP, ADP, AMP), but not adenosine and adenine were elevated in SCT plasma (Figure 5). The latter observation could be at least in part explained by increases in the levels of deaminated purines, including inosine and its catabolites, xanthine and allantoin in SCT plasma (Figure 5). On the other hand, decreases in hypoxanthine and insignificant increases in median circulating levels of urate were observed in SCT subjects (Figure 5).

In keeping with the elevation in circulating levels of monocarboxylates (pyruvate, lactate) in SCT plasma, also tri- (citrate) and dicarboxylates (alpha-ketoglutarate, succinate, fumarate, malate, 2-hydroxyglutarate, itaconate and pyrroline carboxylate) were found to be increased in SCT samples compared to controls (Figures 6.A–B). Insignificant decreases in the levels of glutamine were observed in SCT plasma vs. controls, while significant elevation in plasma phosphocreatine was observed in SCT subjects (Figure 6).

Similarly, tryptophan and most of its metabolites via the kynurenine pathway (kynurenic acid, picolinic acid) or indole metabolism (indole, indoxyl) were elevated in plasma from SCT samples (Figure 7A). In mature RBCs, S1P participates in the stabilization of deoxyhemoglobin at the membrane (Sun et al., 2016), a phenomenon that promotes sickling in the context of SCD (Sun et al., 2017b). S1P and its substrate (sphingosine) or catabolites (phosphoethanolamine, glycerophosphoethanolamine) were all significantly elevated in SCT plasma compared to controls. Elevated levels of plasma N-acetylneuraminate in SCT (Figure 7) is consistent with elevated oxidant damages in SCT individuals (Figure 1) and suggestive of increased membrane damage, leading to desialyation (Li et al., 2021).

### 3.4 Acyl-Carnitines, Fatty Acids and Oxylipins Are Indicative of Increased Membrane Damage in SCT Subjects and Correlate With Impaired Cardiovascular and Kidney Function

Consistent with increased membrane lipid damages and activation of the Lands cycle in individuals with SCT (Figure 8A), elevated plasma acyl-carnitines (Figure 8B) and free fatty acids (Figure 8C) were accompanied by increases in the circulating levels of Coenzyme A precursors (pantothenate), oxylipins (hydroxyeicosatetraenoic—HETEs; hydroxyoctadececnoic—HODE; hydroperoxyeicosatetraenoic—HPETE) and prostaglandins (especially F2α and dinor- F2α) compared to controls (Figure 8C).

## 4 Discussion

Here we report that the plasma metabolome of individuals with SCT is significantly different from the circulating metabolome of healthy subjects from Senegal, thereby providing valuable information about an under-represented population in academic research. Observed changes include an elevation in the plasma levels of mono- (pyruvate, lactate), di- and tricarboxylates (including all Krebs cycle intermediates). Elevation in circulating levels of carboxylic acids in response to hypoxia has been reported in humans and mice following exposure to high-altitude hypoxia (D'Alessandro et al., 2016) or pathological hypoxia secondary (Chouchani et al., 2014) to ischemia or hemorrhage (Peltz et al., 2015). It was reported that individuals with SCT produce more lactate during intense exercise than non-carriers, which is in agreement with the fact anaerobic metabolism could be solicited more in individuals with SCT than in controls to provide ATP for a given stressful situation (Freund et al., 1995). Elevation of monocarboxylates, pyruvate and lactate, have been associated to hypoxia (lactate is the main marker of enhanced lactic anaerobic metabolism in the clinic). More recently, accumulation of RBC and plasma levels of pyruvate have been associated to elevated oxidative stress in the context of glucose 6-phosphate dehydrogenase deficiency (D'Alessandro et al., 2021a), the most common enzymopathy in humans that, like sickle cell-driving mutations, are thought to have evolved to protect from malaria (Luzzatto et al., 2020). Other clinical markers of systemic hypoxia (DʼAlessandro et al., 2017), metabolites like succinate, fumarate and malate have been shown to participate in the stabilization of hypoxia inducible factor 1α and thus promote transcription of its downstream targets, including pro-inflammatory cytokine IL-1β (Tannahill et al., 2013). Here we did not observe significant elevations in circulating levels of interleukins, despite increases in multiple carboxylic acids, suggestive that—in the absence of perturbations that stimulate hypoxia (e.g., exercise, traveling to high-altitude regions) - the mitochondrial dysfunction phenotype associated with SCT may be moderate and homeostatically balanced by organs like the liver that scavenge and metabolize these compounds (Li et al., 2022). Consistently, elevation in the levels of itaconate, a metabolite with potent antinflammatory activity on macrophages (Mills et al., 2018; Ryan et al., 2019), may contribute to explaining the lack of increases in inflammatory cytokines (interleukins and TNFα) in the plasma of individuals with SCT. On the other hand, elevation in plasma levels of kynurenines is suggestive of the activation of interferon responses, which transcriptionally regulate the activity of indole 2,3-dioxygenase, the rate limiting enzyme of this tryptophan metabolic pathway (D'Alessandro et al., 2021b; Thomas et al., 2020b; Cai et al., 2020; Powers et al., 2019). Since this pathway is activated by viral infection via the cGAS-STING response (Di Domizio et al., 2022), and circulating mitochondrial DNA is a DAMP that can stimulate this axis in SCD (Tumburu et al., 2021), it is interesting to speculate that interferon signaling (not tested in this study) may be basally active in SCT, making them more susceptible to untoward inflammatory consequences underlying the etiology of inflammaging associated comorbidities (e.g., anemia of aging, cardiovascular dysfunction) (Franceschi et al., 2018) and the severity of widespread infections like SARS-CoV-2 (D'Alessandro et al., 2021b; Thomas et al., 2020b; Cai et al., 2020; Lawler et al., 2021; Danlos et al., 2021). Individuals with SCT would be at higher risk of SARS-CoV-2-related hospitalization and death than non-carriers (Hoogenboom et al., 2021; Merz et al., 2021) and several cases of vaso-occlusive-like events have been reported in individuals with SCT infected by SARS-CoV-2 ^66,67^. In addition, some kynurenine metabolites that we found to be elevated in SCT individuals (e.g., kynurenic or picolinic acid) are neuroactive metabolites, with a potential role in cognitive impairment (Powers et al., 2019) and nociception (Heyliger et al., 1998), with potential implication in pain crises in SCD patients, which are rarely reported in subjects with SCT. On the other hand, other tryptophan metabolites—indoles—were found to increase in the plasma of individuals with SCT. Though the clinical relevance of this observation is unclear, these metabolites are of bacterial origin and indicate a potential role of microbiome dysbiosis in the etiology of some metabolic alterations in individuals with SCT (Brim et al., 2021).

Markers of hemolysis (biliverdin, bilirubin) were noted to increase in plasma from individuals with SCT, suggestive of basally higher levels of stress to the erythrocyte in these subjects. However, we cannot rule out that any of the SCT subjects was also a carrier of any pro-hemolytic traits that are common in this population, like G6PD deficiency. We previously reported slightly decreased red blood cell deformability in individuals with SCT compared to non-carriers (Tripette et al., 2010), although the magnitude of the decrease is by far less than the one observed in SCD patients (Tripette et al., 2009). Consistently, intracellular glycolytic metabolites, including high-energy phosphate purines (ATP, ADP, AMP) and their deamination products were observed to increase, a phenomenon in part explained by active release of these vasodilatory metabolites and potential activation of RBC AMP deaminase 3 (AMPD3). Indeed, AMPD3 activity is increased in response to oxidative stress (in the protein and lipid fractions—including accumulation of AGEs, AOPP, malondialdehyde, and oxylpins) and decreases in DPG levels (Nemkov et al., 2018), both conditions we observed in the SCT samples tested in this study. Specifically, declines in DPG levels and accumulation of glyceraldehyde 3-phosphate (while inferred from circulating levels of these metabolites and not directly assessed in the cell) are consistent with oxidative stress-induced oxidation of glyceraldehyde 3-phosphate dehydrogenase (GAPDH) (Reisz et al., 2016). Of note, slower GAPDH activity would result in a bottleneck at the triosephosphate isomerase step, with subsequent accumulation of dihydroxyacetone phosphate and its oxidation products, which triggers the activation of glutathione-dependent detoxification through the glyoxylate pathway. Consistently, we observed an elevation in circulating levels of lactoyl-glutathione, a byproduct of this pathway (D'Alessandro et al., 2021a), in plasma from individuals with SCT.

While not an issue in the absence of perturbation in individuals with SCT, elevated basal hemolysis has been associated with increased morbidity in SCD. For example, cardiovascular dysfunction in SCD has been at least in part explained as a function of free hemoglobin NO scavenging (Gladwin et al., 2011; Drawz et al., 2016) or free heme/iron effect on immune cells as result of elevated intra- and extra-vascular hemolysis (Buehler et al., 2021), respectively. In this view, it is interesting to note that extravascular hemolysis is favored by decreases in deformability, previously associated to alterations in acyl-carnitine, free fatty acids and oxylpin levels in the context of oxidant stress after exercise (Nemkov et al., 2021), chronic (Xie et al., 2020; Xu et al., 2022) or acute kidney (Bissinger et al., 2021) dysfunction. While not as sever as previously reported in the context of SCD, here we noted significant increases in the plasma levels of acyl-carnitines and free fatty acids, suggestive of a potential increase in cell vesiculation events and a hallmark of the activation of the Lands cycle, a hallmark of membrane abnormalities in SCD (Wu et al., 2016). Indeed, here we showed that alterations in the levels of oxylipins and acyl-carnitines, as well as of pantothenate (a precursors to CoA). These metabolic changes strongly correlated with blood viscosity, kidney dysfunction (measurements of eGFR and microalbuminuria) and total levels of protein oxidation products. These findings are of particular relevance since it has been demonstrated a 1.5 to 2.0 fold greater risk of chronic kidney disease in individuals with SCT compared to non-carriers (Levey et al., 2009; Tripette et al., 2010; Naik et al., 2014).

This study holds several limitations. First of all, in the present study, most individuals with SCT have HbS of ∼35%, with a smaller population with about ∼25%, almost following a bimodal distribution. This may indicate that 1- and 2-gene-deletion alpha-thalassemia could be quite common in this group. The lower MCV in the SCT group suggests that alpha-thalassemia may be more common, by chance, in the SCT group. This is a potential confounder, especially that cardiovascular effects are studied. However, direct genetic testing of alpha-globin gene status or copy number was not performed owing to logistical limitations. Most of the data interpretation is based on the assessment of circulating plasma levels of selected metabolites that showed a strong correlation with clinically relevant covariates. However, no direct measurements of cellular metabolomes (especially RBCs) was performed in this study, making it impossible to disentangle cell-autonomous from non-cell autonomous contributions to the metabolic derangements we report. Future studies should bridge this gap, while at the same time focusing on the impact of perturbations that may further exacerbate these metabolic changes (e.g., testing the impact of smoking/tobacco use, exercise or exposure to high-altitude hypoxia in individuals with SCT—the latter being recommended against owing to the risk of splenic infarction in these subjects). Previous studies have proposed the use of arginine (Bakshi and Morris, 2016; Morris et al., 2020) or glutamine supplements (Niihara et al., 2018) as a therapy to counteract metabolic aberrations in SCD, though it is unclear whether they would prevent comorbidities in SCT subjects, whereas unmasked by the aforementioned stresses/perturbations. Comparison of SCT data to SCD patients’ plasma and RBCs could provide further clues to the etiology and relative contribution of metabolic dysfunction to the onset and severity of comorbidities in these populations. Combination of such data to genomics readouts (Drawz et al., 2016) would offer an opportunity for the identification of the genetics underpinnings (beyond the HBB E6V mutation) of deranged metabolism in the SCD and SCT groups.

## Data Availability

The original contributions presented in the study are included in the article/Supplementary Materials, further inquiries can be directed to the corresponding authors.
