# Supplementary figures and images for: Plasma Levels of Acyl-Carnitines and Carboxylic Acids Correlate With Cardiovascular and Kidney Function in Subjects With Sickle Cell Trait
Source: Front Physiol. 2022 Jul 13;13:916197. doi: 10.3389/fphys.2022.916197 (PMC9326174; doi:10.3389/fphys.2022.916197)

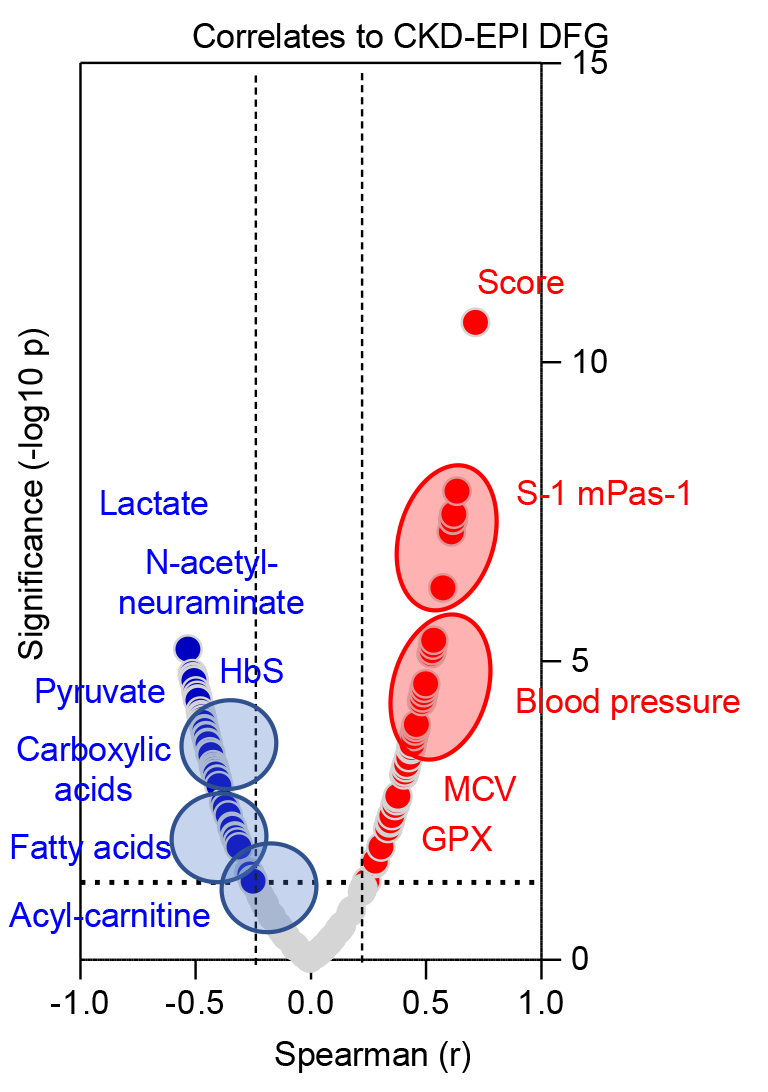

Supplement: Supplementary file 1 [file Image2.TIF]

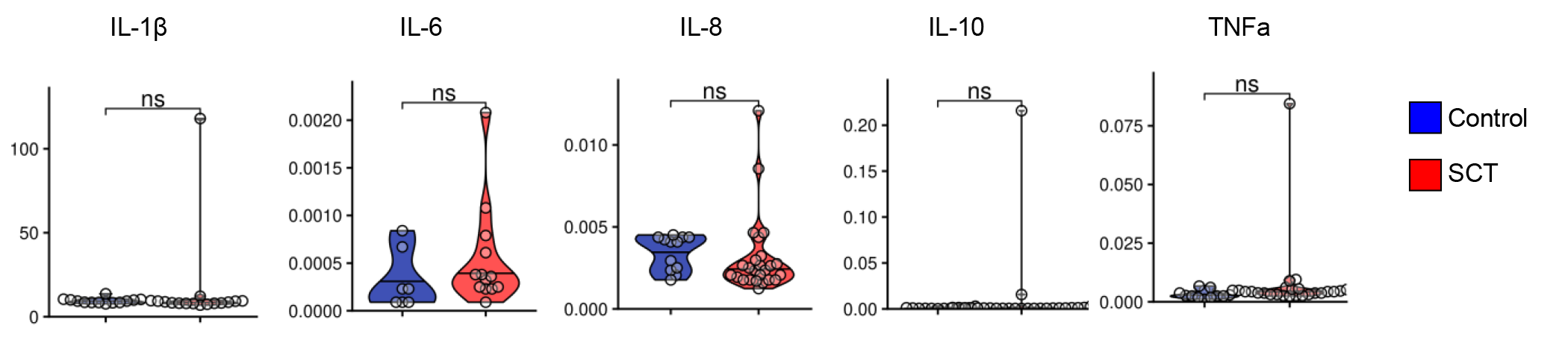

Supplement: Supplementary file 2 [file Image1.TIF]
